# HIV Infection and HIV-Associated Behaviors Among Persons Who Inject Drugs — 23 Metropolitan Statistical Areas, United States, 2018

**DOI:** 10.15585/mmwr.mm7042a1

**Published:** 2021-10-22

**Authors:** Senad Handanagic, Teresa Finlayson, Janet C. Burnett, Dita Broz, Cyprian Wejnert, National HIV Behavioral Surveillance Study Group, National HIV Behavioral Surveillance Study Group, Meaghan Abrego, Alia Al-Tayyib, Bridget Anderson, Narquis Barak, Jeremy M. Beckford, Amisha Bhattari, Kathleen A. Brady, Meredith Brantley, Sarah Braunstein, Sidney Carrillo, Onika Chambers, Thomas Clyde, Sandra Miranda De León, Jie Deng, Rose Doherty, Anna Flynn, Colin Flynn, David Forrest, Danielle German, Sara Glick, Vivian Griffin, Emily Higgins, Abdel R. Ibrahim, Tom Jaenicke, Antonio D. Jimenez, Salma Khuwaja, Jennifer Kienzle, Monina Klevens, Jessica Lin, Zaida Lopez, Yingbo Ma, Christopher Mathews, Jack Marr, María Pabón Martínez, Willi McFarland, David Melton, Timothy W. Menza, Desmond Miller, Luis Moraga, Willie Nixon, Chrysanthus Nnumolu, Conall O’Cleirigh, Jenevieve Opoku, E. Roberto Orellana, Paige Padgett, Jonathon Poe, Marisa Ramos, Toyah Reid, Alexis Rivera, William T. Robinson, Yadira Rolón-Colón, Corrine Sanger, Hugo Santacruz, Ekow Kwa Sey, Jennifer Shinefeld, Daniel Shodell, Brandie Smith, Emma Spencer, Ashley Tate, New York, Jeff Todd, Afework Wogayehu, Pascale Wortley, Margaret Vaaler

**Affiliations:** 1 Division of HIV Prevention, National Center for HIV, Viral Hepatitis, STD, and TB Prevention, CDC.; Nassau-Suffolk, New York; Denver, Colorado; Nassau-Suffolk, New York; New Orleans, Louisiana; New Orleans, Louisiana; Portland, Oregon; Philadelphia, Pennsylvania; Memphis, Tennessee; New York City, New York; New York City, New York; San Diego, California; Chicago, Illinois; San Juan, Puerto Rico; Dallas, Texas; Boston, Massachusetts; San Diego, California; Baltimore, Maryland; Miami, Florida; Baltimore, Maryland; Seattle, Washington; Detroit, Michigan; Detroit, Michigan; Newark, New Jersey; Seattle, Washington; Chicago, Illinois; Houston, Texas; Virginia Beach, Virginia; Boston, Massachusetts; San Francisco, California; Houston, Texas; Los Angeles, California; Memphis, Tennessee; Memphis, Tennessee; San Juan, Puerto Rico; San Francisco, California; Atlanta, Georgia; Portland, Oregon; San Francisco, California; Newark, New Jersey; Miami, Florida; Philadelphia, Pennsylvania; Boston, Massachusetts; Washington, District of Columbia; Portland, Oregon; Houston, Texas; Dallas, Texas; San Diego, California; Virginia Beach, Virginia; New York City, New York; New Orleans, Louisiana; San Juan, Puerto Rico; Detroit, Michigan; Los Angeles, California; Los Angeles, California; Philadelphia, Pennsylvania; Denver, Colorado; Virginia Beach, Virginia; Miami, Florida; Nassau-Suffolk; Atlanta, Georgia; Newark, New Jersey; Atlanta, Georgia; Dallas, Texas

In the United States, 10% of HIV infections diagnosed in 2018 were attributed to unsafe injection drug use or male-to-male sexual contact among persons who inject drugs (PWID) (*1*). In 2017, among PWID or men who have sex with men and who inject drugs (MSM-ID), 76% of those who received a diagnosis of HIV infection lived in urban areas* (*2*). To monitor the prevalence of HIV infection and associated behaviors among persons who reported injecting drugs in the past 12 months, including MSM-ID, CDC’s National HIV Behavioral Surveillance (NHBS) conducts interviews and HIV testing among populations of persons at high risk for HIV infection (MSM, PWID, and heterosexually active adults at increased risk for HIV infection) in selected metropolitan statistical areas (MSAs) (*3*). The estimated HIV infection prevalence among PWID in 23 MSAs surveyed in 2018 was 7%. Among HIV-negative PWID, an estimated 26% receptively shared syringes and 68% had condomless vaginal sex during the preceding 12 months. During the same period, 57% had been tested for HIV infection, and 55% received syringes from a syringe services program (SSP). While overall SSP use did not significantly change since 2015, a substantial decrease in SSP use occurred among Black PWID, and HIV prevalence among Black PWID was higher than that among Hispanic and White PWID. These findings underscore the importance of continuing and expanding HIV prevention programs and community-based strategies for PWID, such as those provided by SSPs, especially following service disruptions created by the COVID-19 pandemic (*4*). Efforts are needed to ensure that PWID have low-barrier access to comprehensive and integrated needs-based SSPs (where legally permissible) that include provision of sterile syringes and safe syringe disposal, HIV and hepatitis C virus (HCV) testing and referrals to HIV and HCV treatment, HIV preexposure prophylaxis, and treatment for substance use and mental health disorders.

In 2018, NHBS staff in 23 MSAs^†^ collected cross-sectional behavioral survey data and conducted HIV testing among PWID; participants were recruited by respondent-driven sampling^§^ (*5*). Eligible participants^¶^ completed a standardized behavioral questionnaire administered in person by trained interviewers. All participants were offered anonymous HIV testing.** Incentives were offered for completing the interview, receiving HIV testing, and recruiting additional participants.^††^ Participants were asked about high-risk HIV acquisition behaviors in the previous 12 months, including receptive sharing of syringes and injection equipment^§§^ or high-risk sexual behaviors,^¶¶^ as well as testing for HIV and HCV infection, participation in HIV behavioral interventions,*** and receipt of syringes from SSPs^†††^ and other sources. Because knowledge of personal HIV infection status could influence risk behaviors, analysis of behavioral data was limited to HIV-negative PWID.^§§§^ Nonheterosexual sexual behavior is not reported in the analysis of high-risk behaviors because the number of HIV-negative MSM-ID in the sample was too small to produce reliable weighted estimates across all 23 MSAs. Data from each MSA were analyzed by using RDS Analyst version 0.7, producing estimates adjusted for peer-recruitment patterns and reported network size along with estimated 95% confidence intervals (CIs) (*5*). To calculate aggregated prevalence of HIV and selected behaviors that are generalizable to PWID across the 23 MSAs, NHBS used a weighted average of MSA-level estimates adjusted for the projected size of the population of PWID in each MSA (*6*).^¶¶¶^ Comparisons were considered significant if there was no overlap in their 95% CIs. This activity was reviewed by CDC and was conducted consistent with applicable federal law and CDC policy.****

In 2018, 14,716 persons were recruited to participate in NHBS; 3,138 (21%) were ineligible, and 230 (2%) were excluded because data were incomplete.^††††^ Among the 11,348 PWID who were tested for HIV, 731 (6%) received positive test results and 10,617 (94%) received negative results (Table 1). Weighted HIV prevalence in the 23 MSAs was 7%, with the highest prevalences among MSM-ID (25%), PWID aged 40–49 years (12%), and Black or African American (Black) PWID (12%). HIV prevalence among Black PWID was higher than that among Hispanic (7%) and White (5%) PWID.

**TABLE 1:** HIV prevalence among persons who inject drugs, by selected characteristics — National HIV Behavioral Surveillance, 23 Metropolitan Statistical Areas, United States, 2018

Characteristic	Total*		HIV-infected*	
	No.^†^	Column % (95% CI)	No.^†^	Row % (95% CI)
**Total**	**11,348**	**100**	**731**	**7 (6–9)**
**Gender**				
Male	7,826	67 (65–69)	500	7 (6–8)
Female	3,425	32 (30–34)	204	8 (5–11)
Transgender	97	1.0 (0.7–1.3)	27	—^§^
**Race/Ethnicity**				
Black, non-Hispanic	3,745	32 (30–34)	335	12 (9–14)
Hispanic^¶^	2,358	24 (22–26)	188	7 (5–8)
White, non-Hispanic	4,458	42 (40–43)	171	5 (4–6)
Other**	189	2 (1–2)	12	—
**Age group, yrs**				
18–29	1,618	15 (14–17)	63	4 (3–6)
30–39	2,999	23 (21–25)	138	5 (4–6)
40–49	2,631	24 (22–25)	201	12 (8–15)
≥50	4,100	38 (36–40)	329	8 (6–10)
**Injection duration**				
≤5 years	2,073	20 (18–21)	77	5 (3–7)
>5 years	9,207	80 (79–82)	647	8 (7–10)
**Education**				
Less than high school diploma	3,240	29 (27–30)	240	8 (6–10)
High school diploma	4,689	42 (40–44)	310	9 (6–11)
More than high school diploma	3,416	30 (28–31)	181	6 (5–8)
**Currently insured**				
No	2,940	18 (16–19)	151	5 (4–7)
Yes	8,362	82 (81–84)	580	8 (6–10)
**Federal poverty level^††^**				
Above federal poverty level	2,771	25 (23–27)	134	7 (5–9)
At or below federal poverty level	8,505	75 (73–77)	596	8 (6–9)
**Drug injected most frequently**				
Heroin only	6,031	55 (53–56)	282	6 (4–7)
Other/Multiple^§§^	5,273	45 (44–47)	444	10 (8–12)
**Male-to-male sex, last 12 months (among males only)^¶¶^**				
Yes	753	10 (8–12)	151	25 (19–30)
No	7,067	90 (88–92)	349	5 (4–6)
U.S. Census region***				
Northeast	2,257	36 (22–49)	180	10 (7–14)
South	4,650	29 (16–42)	365	9 (7–11)
Midwest	1,062	8 (0–21)	17	1 (0–2)
West	2,888	26 (12–39)	112	4 (3–5)

**Abbreviations**: CI = confidence interval; MSA = metropolitan statistical area.

* Aggregate estimates are weighted averages of MSA-level percentages. MSA-level percentages were adjusted for differences in recruitment and the size of participant peer networks of persons who inject drugs, then proportionally weighted by the size of the population of persons who inject drugs in each MSA. MSA-level estimates with insufficient data for analysis were excluded from the aggregated estimates. Aggregated estimates are included in the tables only if at least 15 out of 23 MSA-level estimates were included in the analysis. The average number of MSA-level estimates included in the aggregated estimates for each variable is 21.3.

^†^ Unweighted numbers. Not all categories sum to 11,348 because of missing data.

^§^ Insufficient data to calculate estimates.

^¶^ Hispanic persons might be of any race or combination of races.

** Includes American Indian or Alaska Native, Asian, Native Hawaiian or Other Pacific Islander, and persons of multiple races.

^††^ Poverty level is based on household income and household size.

^§§^ Other drugs injected alone or two or more drugs injected with the same frequency.

^¶¶^ Ascertainment of male-to-male anal sexual contact was restricted to males and includes both insertive and receptive anal sexual contact.

*** *Northeast:* Boston, Massachusetts; Nassau-Suffolk, New York; New York City, New York; Newark, New Jersey; and Philadelphia, Pennsylvania. *South:* Atlanta, Georgia; Baltimore, Maryland; Dallas, Texas; Houston, Texas; Memphis, Tennessee; Miami, Florida; New Orleans, Louisiana; Virginia Beach, Virginia; and Washington, District of Columbia. *Midwest:* Chicago, Illinois and Detroit, Michigan. *West:* Denver, Colorado; Los Angeles, California; Portland, Oregon; San Diego, California; San Francisco, California; and Seattle, Washington. San Juan, Puerto Rico was not included in any of the Census regions.

Among HIV-negative PWID, 26% receptively shared syringes, 68% had condomless vaginal sex, 23% had condomless heterosexual anal sex, 72% had either condomless heterosexual sex or shared syringes, and 43% had more than one opposite sex partner (Table 2). Receptive syringe sharing was higher among White (36%) than among Hispanic (22%) or Black (16%) PWID. Condomless vaginal sex was higher among White (73%) than among Hispanic (63%) or Black (63%) PWID, and condomless heterosexual anal sex was higher among Hispanic (30%) and White (24%) than among Black PWID (16%).

**TABLE 2:** Estimated percentage* of persons who inject drugs who received negative HIV test results and engaged in behaviors^†^ associated with HIV infection in the preceding 12 months, by selected characteristics — National HIV Behavioral Surveillance, 23 Metropolitan Statistical Areas, United States, 2018

Characteristic	% (95% CI)							
	Receptive syringe sharing^†^	Receptive injection equipment sharing^†^	Vaginal sex	Condomless vaginal sex^†^	Heterosexual anal sex	Condomless heterosexual anal sex^†^	Condomless heterosexual sex^†^ or receptive syringe sharing	More than one opposite sex partner
**Total**	**26 (25–28)**	**49 (47–51)**	**77 (75–79)**	**68 (66–70)**	**29 (27–31)**	**23 (22–25)**	**72 (70–74)**	**43 (41–46)**
**Sex**								
Male	24 (22–26)	48 (46–50)	75 (72–77)	64 (61–66)	28 (26–30)	21 (20–23)	69 (67–72)	41 (39–44)
Female	31 (28–34)	50 (47–54)	81 (78–84)	76 (73–79)	32 (28–35)	27 (24–31)	78 (75–81)	48 (44–51)
**Race/Ethnicity** ^§^								
Black, non-Hispanic	16 (14–18)	38 (35–41)	75 (72–78)	63 (60–66)	23 (20–25)	16 (14–18)	66 (63–69)	43 (40–46)
Hispanic^¶^	22 (19–25)	46 (41–51)	73 (68–77)	63 (58–68)	37 (33–42)	30 (26–33)	67 (62–72)	41 (36–45)
White, non-Hispanic	36 (34–39)	59 (56–62)	80 (78–83)	73 (70–75)	29 (26–32)	24 (22–27)	78 (76–81)	45 (42–49)
**Age group, yrs**								
18–29	41 (36–46)	60 (55–65)	89 (86–92)	84 (81–88)	36 (31–41)	30 (26–35)	87 (84–90)	59 (53–64)
30–39	33 (29–36)	54 (50–57)	86 (84–89)	78 (75–81)	34 (31–37)	29 (26–32)	83 (80–86)	50 (47–54)
40–49	23 (20–26)	49 (45–54)	77 (73–81)	68 (64–72)	32 (28–36)	25 (22–29)	72 (68–76)	43 (39–47)
≥50	18 (16–20)	41 (38–44)	66 (63–70)	55 (52–58)	22 (20–25)	16 (14–18)	60 (57–63)	35 (32–37)
**Education**								
Less than high school diploma	25 (22–28)	48 (44–51)	74 (70–77)	64 (60–68)	30 (27–34)	23 (20–26)	70 (66–73)	40 (37–44)
High school diploma	27 (25–30)	49 (46–52)	76 (73–79)	67 (63–70)	28 (25–31)	23 (20–25)	71 (68–74)	44 (41–47)
More than high school diploma	27 (24–29)	50 (46–53)	81 (78–84)	72 (69–75)	30 (27–33)	24 (21–27)	75 (71–78)	46 (43–50)
**Currently insured**								
No	32 (29–35)	49 (46–53)	79 (76–83)	72 (68–75)	30 (27–33)	26 (22–29)	76 (73–80)	50 (46–54)
Yes	25 (23–27)	49 (46–51)	76 (74–78)	67 (64–69)	29 (27–31)	23 (21–24)	71 (69–73)	42 (40–45)
Federal poverty level**								
Above federal poverty level	26 (23–29)	49 (45–53)	83 (80–86)	74 (70–78)	28 (24–32)	22 (19–25)	77 (74–81)	45 (41–49)
At or below federal poverty level	26 (25–28)	49 (47–51)	75 (73–77)	66 (63–68)	30 (28–32)	24 (22–26)	70 (68–73)	43 (41–45)
**Drug injected most frequently**								
Heroin only	26 (24–28)	49 (47–51)	75 (72–77)	66 (63–68)	25 (23–27)	19 (17–21)	70 (67–73)	38 (36–41)
Other/Multiple^††^	27 (25–29)	50 (47–53)	79 (77–82)	70 (67–73)	34 (32–37)	28 (25–31)	74 (72–77)	50 (47–53)
**U.S. Census region^§§^**								
Northeast	27 (24–30)	50 (46–54)	78 (75–82)	70 (66–74)	37 (33–41)	29 (26–33)	73 (69–77)	45 (41–50)
South	28 (25–30)	50 (47–54)	78 (76–81)	69 (66–72)	25 (22–28)	19 (17–21)	75 (72–78)	43 (40–47)
Midwest	21 (17–25)	36 (32–41)	74 (69–78)	60 (56–65)	19 (15–22)	14 (11–17)	64 (59–69)	35 (30–39)
West	25 (22–28)	49 (45–53)	74 (70–78)	65 (61–69)	26 (23–29)	21 (18–24)	69 (65–74)	44 (40–48)

**Abbreviations**: CI = confidence interval; MSA = metropolitan statistical area.

*Aggregate estimates are weighted averages of MSA level percentages. MSA-level percentages were adjusted for differences in recruitment and the size of participant peer networks of persons who inject drugs, then proportionally weighted by the size of the population of persons who inject drugs in each MSA. The average number of MSA-level estimates included in the aggregated estimates for each variable is 22.8.

^†^ Receptive syringe sharing was defined as using needles that someone else had already used to inject with, and receptive injection equipment sharing was defined as using equipment such as cookers, cottons, or water used to rinse needles or prepare drugs that someone else had already used. Condomless vaginal or anal sex was defined as sex without a condom.

^§^ Aggregate estimates for “Other” race and ethnicity (American Indian or Alaska Native, Asian, Native Hawaiian or Other Pacific Islander, and person of multiple races) are excluded because of insufficient data.

^¶^ Hispanic persons might be of any race or combination of races.

** Poverty level is based on household income and household size.

^††^ Other drugs injected alone or two or more drugs injected with the same frequency.

^§§^ *Northeast:* Boston, Massachusetts; Nassau-Suffolk, New York; New York City, New York; Newark, New Jersey; and Philadelphia, Pennsylvania. *South:* Atlanta, Georgia; Baltimore, Maryland; Dallas, Texas; Houston, Texas; Memphis, Tennessee; Miami, Florida; New Orleans, Louisiana; Virginia Beach, Virginia; and Washington, District of Columbia. *Midwest:* Chicago, Illinois and Detroit, Michigan. *West:* Denver, Colorado; Los Angeles, California; Portland, Oregon; San Diego, California; San Francisco, California; and Seattle, Washington. San Juan, Puerto Rico was not included in any of the Census regions.

In the previous 12 months, among HIV-negative PWID, 57% received an HIV test, 33% participated in an HIV behavioral intervention, 55% received syringes from SSPs, and 56% used medication for opioid use disorder (Table 3). Among PWID who were HIV-negative, 83% reported having had a test for HCV in their lifetime and 46% reported being HCV-positive. Fewer White PWID were tested for HIV in the preceding 12 months (53%) than were Hispanic (62%) PWID. Fewer Black PWID received syringes from SSPs (40%) than did Hispanic (63%) or White PWID (63%) or used medication for opioid use disorder (47% versus 65% and 58%, respectively). More PWID with health insurance were tested for HIV infection in the previous 12 months (59%), participated in HIV behavioral interventions (35%), ever tested for HCV infection (86%), and received medication for opioid use disorder (61%) than did PWID without health insurance (47%, 22%, 71%, and 35%, respectively) (Table 3).

**TABLE 3:** Estimated percentage* of persons who inject drugs who received negative HIV test results and participation in testing or prevention services, by selected characteristics — National HIV Behavioral Surveillance, 23 Metropolitan Statistical Areas, United States, 2018

Characteristic	Participation, % (95% CI)						
	Tested for HIV infection in past 12 months	Participated in HIV behavioral intervention in past 12 months^†^	Ever tested for hepatitis C	Self-reported positive for hepatitis C	Received sterile syringes from SSP in past 12 months^§^	Received sterile syringes from pharmacy in past 12 months^§^	Used medication to treat opioid use disorder in past 12 months^¶^
**Total**	**57 (55–59)**	**33 (31–35)**	**83 (82–85)**	**46 (44–49)**	**55 (53–57)**	**36 (34–38)**	**56 (54–58)**
**Gender**							
Male	56 (54–58)	32 (30–35)	82 (80–84)	47 (44–49)	53 (50–55)	35 (32–37)	56 (53–58)
Female	59 (56–62)	33 (29–36)	86 (84–88)	46 (43–50)	61 (58–64)	38 (34–41)	58 (54–61)
Race/Ethnicity**							
Black, non-Hispanic	59 (55–62)	34 (31–37)	80 (78–82)	39 (36–42)	40 (37–42)	20 (17–23)	47 (44–50)
Hispanic^††^	62 (58–66)	37 (33–42)	85 (82–87)	51 (47–55)	63 (58–68)	33 (29–38)	65 (61–69)
White, non-Hispanic	53 (50–56)	29 (27–32)	86 (84–89)	51 (48–54)	63 (60–65)	46 (43–49)	58 (55–61)
**Age group, yrs**							
18–29	59 (54–65)	28 (23–33)	74 (69–79)	29 (24–34)	60 (56–65)	52 (47–56)	52 (47–57)
30–39	60 (56–63)	31 (28–34)	86 (85–88)	43 (40–46)	61 (58–65)	43 (39–46)	61 (57–64)
40–49	60 (57–64)	39 (34–43)	86 (83–88)	49 (45–54)	63 (58–67)	35 (31–39)	60 (56–64)
≥50	52 (49–55)	31 (28–34)	84 (82–87)	54 (50–57)	46 (43–49)	25 (22–27)	52 (49–55)
**Education**							
Less than high school diploma	59 (55–62)	33 (29–37)	84 (81–86)	51 (47–55)	54 (50–58)	27 (24–30)	59 (55–62)
High school diploma	57 (54–60)	31 (28–34)	82 (79–84)	45 (41–48)	55 (52–57)	37 (34–40)	54 (51–57)
More than high school diploma	55 (52–59)	34 (31–37)	86 (84–88)	45 (41–48)	56 (52–59)	42 (38–45)	56 (53–59)
**Health insurance**							
No	47 (43–51)	22 (19–25)	71 (68–75)	30 (26–33)	40 (37–43)	36 (32–40)	35 (31–38)
Yes	59 (57–61)	35 (33–37)	86 (84–88)	50 (48–53)	58 (56–60)	36 (33–38)	61 (59–64)
**Federal poverty level^§§^**							
Above federal poverty level	52 (48–56)	30 (27–34)	82 (79–86)	43 (39–47)	53 (49–56)	48 (43–52)	53 (49–57)
At or below federal poverty level	58 (56–61)	34 (31–36)	84 (82–85)	48 (45–50)	55 (53–57)	32 (30–34)	57 (55–59)
**Drug injected most frequently**							
Heroin only	55 (52–57)	31 (29–34)	85 (83–86)	47 (44–50)	57 (55–59)	37 (35–40)	62 (59–64)
Other/Multiple^¶¶^	61 (58–63)	34 (31–37)	82 (80–85)	47 (44–50)	52 (49–55)	33 (31–36)	51 (48–53)
U.S. Census region***							
Northeast	65 (62–69)	43 (39–47)	88 (85–91)	57 (53–62)	64 (60–68)	37 (33–41)	69 (65–73)
South	57 (54–61)	29 (26–32)	80 (77–82)	39 (36–42)	37 (34–39)	28 (25–31)	46 (43–49)
Midwest	50 (46–55)	28 (24–32)	81 (77–85)	36 (31–41)	43 (38–48)	38 (33–42)	58 (53–62)
West	48 (44–51)	23 (20–26)	84 (80–87)	44 (40–48)	67 (63–71)	42 (38–46)	51 (47–55)

**Abbreviations**: CI = confidence interval; MSA = metropolitan statistical area; SSP = syringe services program.

* Aggregate estimates are weighted averages of MSA-level percentages. MSA-level percentages were adjusted for differences in recruitment and the size of participant peer networks of persons who inject drugs, then proportionally weighted by the size of the population of persons who inject drugs in each MSA. The average number of MSA-level estimates included in the aggregated estimates for each variable is 22.9.

^†^ Participating in an individual or group HIV behavioral intervention (e.g., a one-on-one conversation with a counselor or an organized discussion regarding HIV prevention) did not include counseling received as part of an HIV test or conversations with friends.

^§^ Receiving a syringe from an SSP was defined as reporting receiving a sterile syringe or needles at least once from an SSP or syringe/needle exchange program. Receiving a syringe from a pharmacy was defined as reporting receiving a sterile syringe or needles at least once from a pharmacy.

^¶^ Includes treatment with methadone, buprenorphine, Suboxone or Subutex in the past 12 months.

** Aggregate estimates for “Other” race and ethnicity (American Indian or Alaska Native, Asian, Native Hawaiian or Other Pacific Islander, and person of multiple races) are excluded because of insufficient data.

^††^ Hispanic persons might be of any race or combination of races.

^§§^ Poverty level is based on household income and household size.

^¶¶^ Other drugs injected alone or two or more drugs injected with the same frequency.

*** *Northeast:* Boston, Massachusetts; Nassau-Suffolk, New York; New York City, New York; Newark, New Jersey; and Philadelphia, Pennsylvania. *South:* Atlanta, Georgia; Baltimore, Maryland; Dallas, Texas; Houston, Texas; Memphis, Tennessee; Miami, Florida; New Orleans, Louisiana; Virginia Beach, Virginia; and Washington, District of Columbia. *Midwest:* Chicago, Illinois and Detroit, Michigan. *West:* Denver, Colorado; Los Angeles, California; Portland, Oregon; San Diego, California; San Francisco, California; and Seattle, Washington. San Juan, Puerto Rico was not included in any of the Census regions.

## Discussion

This report provides updated weighted prevalence estimates of HIV infection and behaviors associated with HIV infection since the last NHBS survey among PWID in 2015 (*3*) and represents a snapshot of the HIV prevention landscape for U.S. PWID before the COVID-19 pandemic. In 2018, PWID reported injection and sexual behaviors that placed them at increased risk for HIV infection, highlighting the need for effective and comprehensive prevention services, including access to sterile injection equipment.

From 2015 to 2018, HIV prevalence among PWID in selected MSAs was unchanged at 7%. This analysis found a higher HIV prevalence among Black PWID than among Hispanic or White PWID, despite fewer reported risk behaviors associated with HIV infection among Black PWID. In 2018, when compared with Hispanic or White PWID, fewer Black PWID shared syringes or injection equipment and had condomless anal sex. Overall, SSP use did not significantly increase since 2015 (from 52% to 55%), but a substantial decrease in SSP use among Black PWID (from 51% to 40%), and significantly lower use of SSPs in 2018 among Black PWID compared with Hispanic and White PWID was observed. Lower SSP use among Black PWID in the context of disproportionally higher rates of HIV diagnoses in Black communities (*1*) might lead to increased risk for HIV transmission among Black PWID. It is critical to explore and address the causes for these disparities in SSP use and HIV infection rates.

In 2020, the COVID-19 pandemic impeded delivery of prevention services for PWID nationally, resulting in a substantial reduction in SSP operations and provision of medication for opioid use disorder (*4*). This analysis highlights the ongoing need for risk reduction and improved access to HIV prevention services among PWID than existed before the COVID-19 pandemic, especially because access to these services was reduced as a result of the pandemic. Findings from this analysis and continuous monitoring of characteristics and risk behaviors associated with HIV infection of PWID will facilitate estimation of how the pandemic disrupted behaviors as well as access to essential prevention services among PWID.

The findings in this report are subject to at least four limitations. First, because a method of obtaining standard probability-based samples of PWID does not exist, the representativeness of the NHBS sample cannot be determined. Although adjustments were made to the sampling methodology (*5*), biases related to participants’ recruitment behavior or their willingness and ability to participate in the interview might have affected the sample. Second, insufficient numbers of participants in some cities precluded inclusion of these cities in the aggregate estimates. The number of MSAs excluded from aggregate estimates varied based on the analysis variable. Third, PWID were interviewed in 23 MSAs with high prevalences of HIV infection; findings from these MSAs might not be generalizable to all PWID, including residents of rural or nonmetropolitan areas. Finally, behavioral data are self-reported and subject to recall and social desirability biases.

Despite decades of evidence regarding the importance of SSPs and regular HIV testing for the prevention of HIV transmission among PWID (*7*,*8*), only approximately one half of PWID used SSPs or were tested for HIV in the 12 months preceding the survey. Since 2015, the number of SSPs and the number of syringes distributed in the United States increased (*9*); however, this analysis found no significant increase in the overall use of SSPs and a substantial reduction in SSP use among Black PWID compared with 2015. The ongoing drug-use epidemic has increased the potential for HIV outbreaks among PWID, particularly in areas and among groups that have limited access to prevention services such as SSPs and medications for opioid use disorder (*10*). For progress to be made toward achieving the goals of the federal Ending the HIV Epidemic in the United States initiative,^§§§§^ PWID need to have low-barrier access to comprehensive and integrated needs-based SSPs (where legally permissible) that include provision of sterile syringes and safe syringe disposal, HIV and HCV testing and referrals to HIV and HCV treatment, HIV preexposure prophylaxis, and treatment for substance use and mental health disorders.

**Summary**

**What is already known about this topic?**

In 2015, the estimated HIV infection prevalence among persons who inject drugs (PWID) in 20 U.S. metropolitan statistical areas was 7%.

**What is added by this report?**

In 2018, estimated HIV prevalence among PWID remained unchanged, and although overall syringe service program use did not significantly change, a substantial decrease in their use occurred among Black PWID.

**What are the implications for public health practice?**

Low-barrier access is needed to comprehensive and integrated needs-based syringe service programs (where legally permissible) that include provision of sterile syringes and safe syringe disposal, HIV and hepatitis C virus testing and referrals for treatment, HIV preexposure prophylaxis, and treatment for substance use and mental health disorders for PWID.

## Footnotes

* Urban areas include metropolitan statistical areas with populations of ≥500,000 persons; areas with populations of <500,000 persons were considered nonurban.

† Atlanta, Georgia; Baltimore, Maryland; Boston, Massachusetts; Chicago, Illinois; Dallas, Texas; Denver, Colorado; Detroit, Michigan; Houston, Texas; Los Angeles, California; Memphis, Tennessee; Miami, Florida; Nassau-Suffolk, New York; New Orleans, Louisiana; New York, New York; Newark, New Jersey; Philadelphia, Pennsylvania; Portland, Oregon; San Diego, California; San Francisco, California; San Juan, Puerto Rico; Seattle, Washington; Virginia Beach, Virginia; Washington, District of Columbia.

§ Recruitment chains in each MSA began with four to 28 initial participants identified during formative assessment (the process by which researchers define a community of interest, determine how to access that community, and describe the attributes of the community that are relevant to a specific public health issue). Initial participants who participated in the survey were asked to recruit up to five other persons who inject drugs using a coded coupon system designed to track referrals. All eligible participants were asked to recruit up to five other persons who inject drugs. Respondent-driven sampling analysis was done using RDS Analyst version 0.7.

¶ Eligible participants were persons who injected drugs that were not prescribed to them by a physician during the previous 12 months, resided in the MSA, were aged ≥18 years, could complete the interview in English or Spanish, and provided informed consent.

** All 23 MSAs conducted HIV screening with a rapid test; for supplemental testing to confirm rapid tests, 19 conducted a second orthogonal rapid test, one collected blood via venipuncture, and three collected blood via dried blood spots. A nonreactive rapid test result was considered HIV-negative, and a reactive rapid test result was considered HIV-positive, if supported by a second rapid test or supplemental laboratory-based testing.

†† The incentive format (cash or gift card) and amount varied by MSA based on formative assessment and local policy. A typical format included $25 for completing the interview, $25 for providing a specimen for HIV testing, and $10 for each successful recruitment (maximum of five).

§§ Receptive sharing of syringes was defined as using needles that someone else had already used to inject with, and receptive sharing of injection equipment was defined as using equipment such as cookers, cottons, or water used to rinse needles or prepare drugs that someone else had already used.

¶¶ Condomless vaginal sex and condomless anal sex were defined as sex without a condom at least once in the past 12 months. Ascertainment of male-to-male anal sexual contact includes both insertive and receptive anal sexual contact.

*** Participating in an individual or group HIV behavioral intervention was defined as a conversation with a counselor or an organized discussion regarding prevention of HIV infection and did not include counseling received as part of an HIV test or conversations with friends.

††† Receiving a syringe from an SSP was defined as receiving a sterile syringe or a needle at least once from a needle or syringe exchange program during the previous 12 months. Medication for opioid use disorder includes treatment with methadone, buprenorphine, and Suboxone or Subutex.

§§§ Behavioral analyses from previous reports (https://doi.org/10.15585/mmwr.mm6701a5) excluded participants reporting a previous HIV-positive test result. A comparison of analysis excluding those who previously had received a positive HIV test result did not yield significantly different estimates.

¶¶¶ For MSA-level estimates for which CIs could not be calculated, maximally wide CIs (0–1) were used in aggregation. MSA-level estimates with insufficient data for analysis were excluded from the aggregated estimates. Aggregated estimates are included in the tables only if ≥15 out of 23 MSA-level estimates were included in the analysis. The highest number of missing MSA-level estimates for one variable was five.

**** 45 C.F.R. part 46.102(l)(2), 21 C.F.R. part 56; 42 U.S.C. Sect. 241(d); 5 U.S.C. Sect. 552a; 44 U.S.C. Sect. 3501 et seq.

†††† Data from 230 participants were excluded because of missing recruitment data, lost data during electronic upload, incomplete survey data, and survey responses with questionable validity or invalid HIV test results. Reasons for exclusion were not mutually exclusive.

§§§§
https://www.hrsa.gov/ending-hiv-epidemic
